# Can we reduce the cardiovascular risk in peritoneal dialysis patients?

**DOI:** 10.4103/0971-4065.65296

**Published:** 2010-04

**Authors:** Y.W. Chiu, R. Mehrotra

**Affiliations:** 1 Division of Nephrology and Hypertension, Los Angeles Biomedical Research Institute at Harbor-UCLA Medical Center, Torrance, CA, U.S.A; 2 Division of Nephrology, Kaohsiung Medical University Hospital, Kaohsiung, Taiwan, CA, U.S.A; 3 David Geffen School of Medicine at UCLA, Los Angeles, CA, U.S.A

**Keywords:** Cardiovascular disease, dyslipidemia, end-stage renal disease, hypertension, infection, mineral metabolism, mortality, peritoneal dialysis

## Abstract

Patients with end-stage renal disease (ESRD), including those treated with peritoneal dialysis (PD), have a high risk for death, particularly from cardiovascular (CV) causes. Traditional risk factors for CV disease – like hypertension, diabetes, and dyslipidemia - are highly prevalent, often severe, and more difficult to treat in dialysis patients. Development of strategies for CV risk reduction in dialysis patients is complicated by epidemiologic studies that demonstrate paradoxical associations of some of the traditional risk factors with mortality. The difficulty is enhanced by either a paucity or negative findings of studies that have tested risk modification by targeting traditional CV risk factors. It is also clear that neither the prevalence nor the severity of traditional risk factors explains the substantial increase in risk for death associated with ESRD; this has led to identification of several nontraditional risk factors. Among these, systemic inflammation, disordered mineral metabolism, and long-term CV risk from infectious complications appear the most promising. However, the evidence in favor of the importance of these risk factors is largely limited to observational studies. In this review, we present a critical analysis of the literature to assist the clinician to reduce the CV risk of ESRD patients treated with PD.

## Introduction

Dialysis patients have a substantial increased risk for death, as reported by the United States Renal Data System (USRDS), the mortality rate of dialysis patients is eightfold higher than in age- and gender-matched controls and in the United States; fewer than a third are alive after 5 years of maintenance dialysis therapy.[1] This high mortality is largely a result of a high prevalence of cardiovascular disease (CVD), the leading cause of death of end-stage renal disease (ESRD) patients. In the United States, more than one-half of incident ESRD patients have some form of CVD (i.e., heart failure, coronary disease, peripheral vascular disease, and cerebrovascular disease) present before the start of renal replacement therapy and newly diagnosed CVD develops at an annual rate of 10% per year.[1] Patients treated with maintenance hemodialysis have twice the incidence of recurrent myocardial infarction and sudden death compared to the general population after the first episode of myocardial infarction.[2–4] Overall, CVD attributes to a half of the total mortality occurring in dialysis patients and the ratio increases with the increase of age.[1]

However, pathogenic mechanisms that lead to CV mortality in dialysis patients may not be quite the same as in the general population. There is strong evidence to suggest that atherogenesis is accelerated in dialysis patients. However, sudden cardiac death is the single most common cause of death among dialysis patients; it is unclear if it is related to coronary atherosclerosis.[1,4] Furthermore, even though risk factors traditionally associated with CVD are commonly present and are often severe in dialysis patients, they are insufficient to explain the substantial increase in CV risk.[5] Furthermore, the paradoxical association of traditional cardiovascular (CV) risk factors with mortality in epidemiologic studies of dialysis patients has led some to question the importance of the same. Several non-traditional risk factors have been identified and are currently under investigation [Table 1]. Non-traditional CV risk factor is a phrase that distinguishes those CV risk factors that were not originally identified in the Framingham study.[6] As expected, some of these CV risk factors interact with each other in pathogenesis of CVD, i.e., fluid overload worsens hypertension and increased oxidative stress enhances inflammation.[7] Given the complicated interactions within and between the above two categories, the relative importance of traditional and non-traditional factor probably varies considerably from one patient to the other. In this review, we present an overview of the literature of the importance of some of these risk factors in dialysis patients, particularly among those treated with peritoneal dialysis (PD), and determine how this knowledge can be used to decrease CV risk of these patients.

**Table 1:** Cardiovascular risk factors in

Traditional CV risk factors	Non-traditional CV risk factors, uremia related
Age	Fluid overloading
Gender (male)	Anemia
Smoking	Oxidative stress
Diabetes mellitus	Mineral metabolism disorder/CV calcification
Hypertension	Inflammation/malnutrition
Hyperlipidemia	Dyslipidemia
Left ventricular hypertrophy	Endothelial dysfunction
Obesity	Autonomic dysfunction
Family history	Hyperhomocysteinemia

## Blood Pressure Control and Volume Management

**Figure 1:**
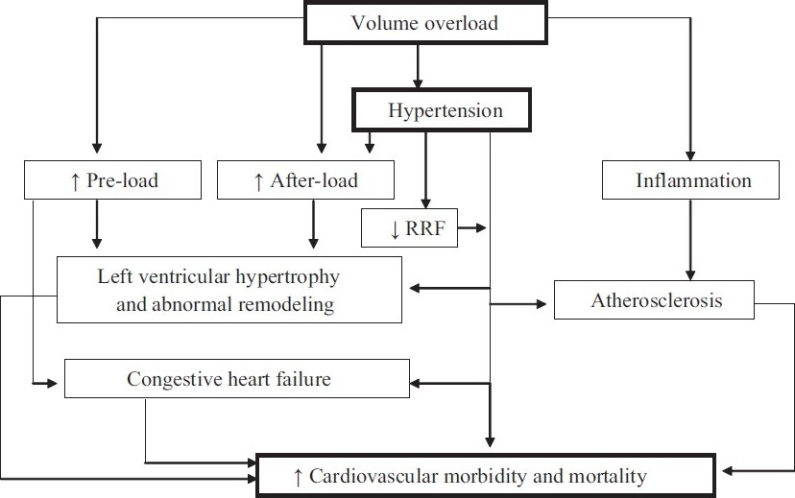
Schematic presentation of fluid overload and CV mortality in PD patients; fluid overload in PD patient can contribute to CV morbidity and mortality through many pathways

Hypertension, a well-recognized traditional CV risk factor, is present in the overwhelming majority of all dialysis patients.[8,9] In addition to the well-recognized effects of hypertension in inducing left ventricular hypertrophy and congestive heart failure and atherosclerosis, in dialysis patients there is additional concern that uncontrolled blood pressure is associated with a more rapid loss of residual renal function and its attendant risk [Figure 1].[10,11] In patients treated with PD, the relationship between systolic blood pressure and mortality is U-shaped, such that both high and low blood pressure have been reported to be associated with increased risk for death. In a study on 125 patients who had survived 6 months after the start of PD, Ates *et al*. reported that every 10 mm Hg higher systolic blood pressure was associated with a 64% higher adjusted risk for death over a 3-year follow-up period.[12] In contrast, in 1059 prevalent PD patients enrolled in Dialysis Morbidity and Mortality Study (DMMS) Wave 2 study of the United States Renal Data System, a higher risk for all-cause and CV mortality over a 3-year follow-up was seen only in individuals with low blood pressure; subgroup analysis showed that the association of lower blood pressure with higher risk for death was significant only in diabetics, those with heart failure, and among those treated with anti-hypertensive medications.[13] A recent study from the United Kingdom has tried to clarify these discordant findings – among 2770 incident PD patients, low blood pressure at baseline was associated with a higher risk for death during the first year of dialysis but higher systolic and pulse pressure was associated with a higher risk for death over 6-years of follow-up.[14] It appears that individuals with low blood pressure at baseline are those who have underlying heart failure or other cardiac co-morbidities and it is the underlying heart disease – and not the low blood pressure – that is the cause for the higher risk for early death. Unfortunately, not enough dialysis patients live long enough for epidemiologic studies to identify the long-term risk associated with uncontrolled hypertension. This possibly explains the paradoxical findings of epidemiologic studies in dialysis patients including among those treated with PD. A recent meta-analysis indicates that treatment of dialysis patients with anti-hypertensive agents is associated with a survival advantage.[15] Indeed, there are no controlled trials that indicate a higher risk for death in dialysis patients in whom therapeutic interventions that lower blood pressure lead to a higher risk for death. These different pieces of evidence suggest that failure to treat hypertension in dialysis patients may compromise the long-term survival of those patients who survive the first few years of dialysis treatment; there seems to be little evidence for harm in treating uncontrolled blood pressure in dialysis patients, including those treated with PD. Consistent with this approach, the Kidney Disease Outcome Quality Initiative (K/DOQI) has recommended to maintain the blood pressure at less than 140/90 mm Hg.[16]

There are three components of the management of hypertension in dialysis patients: dietary salt restriction, correction of volume overload, and drug therapy. In a randomized controlled trial, increase in sodium chloride intake has been shown to result in increase in blood pressure of PD patients. This study reinforces the need to instruct patients to restrict dietary sodium intake to manage hypertension.[17]

In dialysis patients, correction of hypervolemia is important in the management of hypertension [Figure 1]. In addition to inducing hypertension, fluid overload may directly lead to systemic inflammation. Reliable assessment of the volume status of dialysis patient is one of the biggest challenges in achieving euvolemia in dialysis patients. There is no evidence that use of bio-electrical impedance or serum concentration of atrial natriuretic peptides is any superior to routine clinical assessment in improving the clinical outcomes of dialysis patients and cannot be recommended for routine clinical use at this time. High-dose loop diuretics have been shown to maintain urine volume in PD patients, have the potential to simplify volume management of PD patients, and should be considered in every patient with significant residual renal function.[18] However, appropriate management of the long dwell (the overnight dwell in continuous ambulatory PD patients, and the day dwell in automated PD patients) is critical to achieving and maintaining euvolemia. Effective strategies in the management of long dwell include reduction in dwell time (e.g., partially dry day or adding a day exchange during the long dwell in automated PD patients), using of hypertonic dialysate, and/or use of icodextrin. Aggressive ultrafiltration has the potential to achieve normotension in many dialysis patients; caution must be exercised since repeated episodes of hypovolemia have been associated with a more rapid loss of residual renal function in dialysis patients.[11,19]

Despite adequate management of volume status, many patients require drug therapy. There are no controlled trials that have evaluated the effect of angiotensinconverting enzyme inhibitors and angiotensin receptor blockers on fatal and non-fatal CV events in PD patients; the results of the two clinical trials in HD patients were discordant.[20,21] However, the benefit of these drugs in preserving residual renal function in PD patients is well established, and there is preliminary evidence to suggest that these classes of drugs may have salutary effects on maintaining peritoneal membrane function.[22–24] Thus, these classes of drugs should be the first choice for anti-hypertensive therapy. Since there is no potassium in peritoneal dialysate, there is little, if any, risk for hyperkalemia in PD patients treated with these drugs.[25] Use of carvedilol has been shown to reduce risk for death in HD patients with reduced left ventricular systolic function;[26] even though evidence is lacking for PD patients, beta blockers should be additionally considered in patients with left ventricular dysfunction. As recommended by K/DOQI, the decision to select other classes of anti-hypertensive agents may be based or based only on the extrapolation of studies in the general population;[16] the limits of such extrapolation, though, are increasingly being recognized.

## Dyslipidemia

Dyslipidemia in dialysis patients is generally characterized by an elevated total cholesterol, lipoprotein (a), small-dense low-density lipoprotein cholesterol, triglycerides, very low density lipoprotein cholesterol, and decreased high-density lipoprotein cholesterol and apo-A1.[27] There is evidence to suggest that the dyslipidemia in PD patients may be worse than in those treated with HD; this may be secondary to absorption of glucose from the peritoneal dialysate, and a higher prevalence of hypoalbuminemia in PD patients (in part from higher peritoneal protein losses, not unlike that seen in patients with nephritic syndrome). Even though direct evidence is lacking, dyslipidemia may contribute to the accelerated atherogenesis observed in many dialysis patients. However, most of the epidemiologic studies have demonstrated a paradoxical association between lipid parameters and all-cause and CV mortality and non-fatal CV events in both HD and PD patients.[28–30] In addition to selection bias and competing risk factors, further studies implied that this paradoxical association of low serum cholesterol with higher risk for death may be secondary to the confounding influence of inflammation. Among the 823 dialysis patients enrolled in the CHOICE study (19% treated with PD), the paradoxical association of cholesterol with mortality was seen only in subgroup of patients with malnutrition and/or inflammation. In well-nourished patients with no evidence of inflammation, the relationship of total cholesterol to mortality was the same as seen in the general population, i.e., every 40 mg/dl increase in total cholesterol was associated with a 51% higher risk for all-cause mortality and 173% higher risk for CV mortality.[31] Similarly, in the analysis of data from 1053 PD patients enrolled in the DMMS Wave 2 study, the association between low cholesterol and higher risk for death was stronger in participants with serum albumin level less than 3 g/dl.[30] Thus, the confounding influence of wasting accompanying ESRD seems to partially contribute to the paradox.

In PD patients, a small, randomized cross-over study substituting icodextrin for glucose for the long dwell has been shown to lead to a reduction in total and low-density lipoprotein cholesterol.[32] Thus, a reduction in glucose exposure with the use of icodextrin is an important tool in the management of dyslipidemia of PD patients. Statins remain the mainstay of drug therapy of dyslipidemia in dialysis patients. The magnitude of reduction in total and low-density lipoprotein cholesterol with statins in dialysis patients is the same as seen in the general population and there is no increase in risk for adverse events.[27] The most important question, however, is whether correction of dyslipidemia in PD patients can lead to the improvement of CV morbidity and mortality, just as it works in general population. In an observational study using the Dialysis Morbidity and Mortality dataset, incident PD patients treated with lipid lowering agents (93% statins) were seen to have a 26% lower all-cause mortality and 33% lower CV mortality.[33] However, observational clinical trials are often beset by selection bias. Unfortunately, there are no randomized, controlled trials that evaluate the effect of treatment with statins on fatal and non-fatal CV events or all-cause mortality of PD patients. Two randomized controlled trials have evaluated the effect of statins on “hard outcomes” in hemodialysis patients: the 4D (Die Deutsche Diabetes Dialyse Studie; 1255 diabetic HD patients treated with atorvastatin, followed for a median of 4 years), and AURORA (A Study to Evaluate the use of Rosuvastatin in subjects on Regular Dialysis; 2776 HD patients followed for a median of 3.8 years). Despite a significant reduction in cholesterol, these studies were unable to demonstrate a reduction in fatal and non-fatal CV events.[34,35] At least one more clinical trial, SHARP (Study of Heart and Renal Protection; interventions – simvastatin, or simvastatin and ezetimibe, or placebo), has completed recruitment of dialysis and non-dialysis dependent chronic kidney disease (CKD) subjects and its results are awaited.[36]

PD patients are also more likely to have elevated serum triglyceride levels. In an analysis of the DMMS it was found that patients with serum triglycerides > 200 mg/dl had a higher risk for death.[30] Furthermore, extreme elevations of triglyceride levels can lead to pancreatitis. Fibrates and niacin are often used to lower serum triglyceride levels. However, care should be exercised in combining statins with fibrates in dialysis patients since this combination is associated with a higher risk for hepato-toxicity. Use of long-acting preparation of niacin obviates the adverse events of facial flushing seen with the short-acting preparations and appears to be effective in lowering elevated triglyceride levels. Unfortunately, there are no interventional studies that demonstrate a reduction in risk for death with lowering serum triglyceride levels.

To summarize, dialysis patients have an atherogenic lipid profile and this may be worse in patients undergoing PD. Over three-quarters of dialysis patients have malnutrition and/or inflammation and in these patients, epidemiologic studies show a paradoxical association between lipid parameters and outcome. Two large-scale randomized controlled trials have failed to show a survival benefit for hemodialysis patients treated with statins. Given the strong body of data on the protective effect of lipid lowering therapy in the general population, it is difficult to exclude a protective effect of statins for at least some dialysis patients. However, the overall beneficial effect of lipid lowering in dialysis patients may be smaller than previously anticipated. It is also unlikely that a randomized, controlled trial of lipid lowering will be undertaken in PD patients. This leaves us with using our clinical judgment in making therapeutic decisions; we continue to periodically monitor the lipid profile of our PD patients and target therapeutic interventions to achieve a low-density lipoprotein concentration to <100 mg/dl.

## Glycemic Control

It is well established that tight glycemic control can lower the risk for microvascular complications of diabetes mellitus; however, its effect on macrovascular complications remains inconclusive.[37–39] There are several issues that complicate the management. First, assessment of glycemic control is challenging in dialysis patients, particularly in those treated with PD. Inaba *et al*. measured the post-prandial glucose concentrations in 1300 subjects for 3 months. They demonstrated that dialysis patients had lower HbA1c values than that of those without CKD with the same average glucose level, suggesting that HbA1c underestimates the glucose level in dialysis patients.[40] The authors further showed that this underestimation might be secondary to the use of erythropoietin – a larger proportion of circulating erythrocytes in EPO treated patients are younger that have not been around for long enough for sufficient glycoysylation of hemoglobin. They demonstrated a greater predictive value with the use of glycated albumin which measures glycemic control over the preceding 2 weeks and is not affected by serum albumin concentrations.[40] However, the ability of glycated albumin to predict risk for microvascular and macrovascular complications is limited, the assay is not commercially available, and is not ready to be used in clinical practice. In addition to the limitation imposed by the use of HbA1c, PD patients continuously absorb glucose from the PD solution and are never truly in the fasting, or post-absorptive state. Thus, it is important to consider the limitations of both HbA1c and home glucose monitoring in PD patients before one makes therapeutic decisions.

Second, in most populations development of a therapeutic regimen for the management of diabetes mellitus assumes an overnight fasting or post-absorptive state and a day-time post-prandial state. However, these assumptions are often violated in patients treated with PD as there is continuous glucose absorption from the peritoneal cavity; the magnitude of this absorption is significantly greater among patients who are treated with automated PD. Furthermore, the glucose load can vary based upon the dialysate concentration of glucose. This altered physiology needs to be taken into account when designing a therapeutic prescription for the management of diabetes mellitus in PD patients.

Third, in addition to use of subcutaneous insulin, PD patients can be treated with intraperitoneal insulin (only regular insulin can be used for the latter regimen). Intraperitoneal administration has the advantage of obviating the need for injections and the possibility of a tighter control since insulin can be administered with each exchange and thus, more frequently. However, the use of intraperitoneal insulin is more challenging in automated PD patients. Furthermore, regular injection of insulin into the dialysate bag runs the risk of peritonitis from touch contamination. Finally, practitioners who use intraperitoneal insulin should be familiar with a complication, sub-capsular heapatic steatosis. This appears only as a radiologic abnormality with no clinical sequelae.[41]

Finally, the targets for glycemic control in PD patients are currently undefined. Adequate glycemic control is probably desirable to reduce risk for progression of complications like retinopathy. However, the results of the recent clinical trials like the ACCORD argue for caution in targeting for an overly aggressive target.[42] Recognizing the limitations of measurement, our goal is to achieve an HbA1c of <7% in our dialysis patients, including those treated with PD.

In addition to glycemic control in diabetic patients, there is a small but finite incidence of new-onset diabetes mellitus; this incidence appears to be slightly higher in patients treated with PD. Furthermore, recent studies suggest that dialysis patients with morning blood sugar values between 100 and 126 mg/dl have a higher risk for death compared to those whose blood sugars are <100 mg/dl.[43] Thus, routine periodic monitoring of blood sugar concentrations is important even in non-diabetic dialysis patients, and in individuals in whom the blood sugar values are elevated but are not high enough to meet the definition of diabetes mellitus, aggressive life style interventions should be implemented.

## Mineral Metabolism Disorders

Disorders of mineral metabolism, an inevitable consequences of loss of glomerular filtration rate, have now reproducibly been shown to be associated with a higher risk for death in both dialysis and non-dialysis dependent CKD patients.[44] Of the abnormalities in mineral metabolism, the strongest body of data pertains to the risk associated with hyperphosphatemia and vitamin D deficiency.

## Hyperphosphatemia

At least two studies have demonstrated that the higher risk for death with elevated serum P concentrations is seen in PD patients as well.[45,46] There are several mechanisms whereby elevated serum phosphorus can potentially increase the vascular risk of patients. A large body of laboratory data indicates that phosphorus can induce vascular calcification; the severity of vascular calcification has consistently been associated with a higher risk for death.[44] Furthermore, hyperphosphatemia is associated with left ventricular hypertrophy and a more rapid loss of residual renal function, providing an additional basis to explain the higher risk for death.[47,48] To date, there is no study which directly demonstrates that lowering serum P will reduce the mortality of any group of patients, including those treated with PD. Given the compelling nature of laboratory data, it is probably prudent to aggressively manage hyperphosphatemia till such evidence is available. The recently issued Kidney Disease: Improving Global Outcomes (KDIGO) guidelines do not provide firm thresholds but recommend that the goal of treatment should be to normalize serum phosphorus levels.[49] The achievement of this goal, undoubtedly, will be challenging.

As summarized in a recent review on the topic, there are four therapeutic strategies that may help in reducing serum phosphorus levels in PD patients: (1) restriction of dietary phosphorus while ensuring a high protein intake; (2) maintenance of residual renal function; (3) maximization of peritoneal phosphorus clearance; and (4) prescription of phosphate binders.[50] In the United States, the dietary intakes of P have increased over the past 30 years. This has been driven both by an increase in intake of food naturally rich in phosphorus (like meats) and greater use of processed foods, including fast foods. Balancing a high dietary protein with low phosphorus intakes is often challenging but dietary counseling should focus on minimizing the use of two important groups of foods – those with high phosphorus but little, if any, protein (like carbonated drinks), and limiting the use of processed foods as well as fast foods. Both the latter strategies have been shown to significantly lower the serum P in HD patients and it is likely that the same benefit will ensue in PD patients as well.[51]

As discussed earlier, evidence supports the routine use of angiontensin-converting enzyme inhibitors and/or angiotensin receptor blockers for the preservation of residual renal function in PD patients. Furthermore, the risk of hyperkalemia is substantially lower in PD patients, and thus, these therapies are safe for most patients. A typical PD prescription is able to remove about 400 mg of P daily.[52] The prescription can be modified to increase peritoneal P excretion; since the peritoneal P and creatinine clearances are highly correlated, any intervention that increases peritoneal creatinine clearance should be expected to result in an increase in peritoneal P removal.[52,53] This could be achieved by increasing the fill volume, ensuring a continuously wet abdomen, or by adding a day exchange in patients treated with automated PD. Although more rapid nighttime cycling increases the phosphorus clearance, the increase in daily P removal is <50 mg and cannot be recommended.[54]

Despite these maneuvers, most PD patients require P-binders. A detailed discussion of P-binders is beyond the scope of this review. While the low cost makes Ca-based binders attractive, there are enough data to raise concern about a more rapid progression of vascular calcification in dialysis and non-dialysis patients treated with these drugs;[55,56] the data on the effect of calcium avoidance on mortality is, however, inconsistent.[57,58] Even if Ca-based P-binders are used as a first-line therapy for economic reasons, patients who develop hypercalcemia or in whom the parathyroid hormone is over-suppressed should be switched to non-calcium based binders like lanthanum carbonate, or sevelamer hydrochloride or carbonate.

## Hypovitaminosis D

Vitamin D is synthesized in the skin in the presence of ultraviolet light, is 25-hydroxylated in the liver to produce 25-OH vitamin D, and finally 1-α hydroxylated in the kidney to produce the active form of vitamin D, 1,25 di(OH) vitamin D.[59] The half-life of 25-OH vitamin D is substantially longer than that of the active form of vitamin D, and the circulating serum 25-OH vitamin D levels are about a 1000-fold higher.[60] Thus, measurement of serum 25-OH vitamin D is the best way to measure the adequacy of body stores of vitamin D.[60]

Since 1-α hydroxylation is the primary source of circulating active vitamin D, it has long been recognized that patients with CKD have low 1,25 di(OH) vitamin D levels. However, the existing clinical paradigm does not include vitamin D repletion but involves the administration of supra-therapeutic concentrations of active vitamin D only for the treatment of secondary hyperparathyroidism. There is evidence that serum 25-OH vitamin D levels are significantly lower in patients with all stages of CKD, particularly among those treated with PD.[61–63] The higher prevalence of hypovitaminosis D may be related to lower functional capacity of CKD patients (and lower exposure to ultraviolet light), lower dietary intakes, and increased urinary and/or peritoneal losses of vitamin D. Furthermore, a graded relationship exists between serum 25-OH vitamin D levels and all-cause mortality, and fatal and non-fatal CV events at all stages of CKD, including among those treated with PD.[63–65] There is an emerging body of laboratory data that provides a biologic basis to explain the association of hypovitaminosis D with CV risk.[66] For example, there is an inverse association of serum 25-OH vitamin D levels with insulin resistance, and in animal studies administration of paricalcitol is associated with regression of left ventricular hypertrophy. Finally, observational studies have demonstrated that non-dialysis dependent CKD and HD patients treated with active vitamin D have a lower risk for death.[67,68] All these studies raise the question if there is a need to change the clinical paradigm, i.e., rather than using vitamin D therapy to manage secondary hyperparathyroidism, should vitamin D repletion be the goal of therapy irrespective of serum parathyroid hormone levels at all stages of CKD, including dialysis patients? Should repletion occur with calciferols (cholecalciferol or ergocalciferol) or calcitriol or analogs, including among dialysis patients? At this time, there are insufficient data to recommend a change in the clinical paradigm. However, the accumulating data are compelling and call for interventional studies, including among dialysis patients. In the meantime, given evidence for survival benefit, active vitamin D, or the analogs, should probably be the first line therapy for secondary hyperparathyroidism.

## Infectious Complications and Cardiovascular Risk

There is increasing evidence to support that acute infectious complications increase the long-term risk for CV mortality and morbidity of HD patients.[69–71] Consistent with these observations, evidence suggests that acute episodes of peritonitis increase long-term vascular risk. Thus, episodes of gram negative peritonitis are associated with 20% 6-month mortality; less than one-half of these deaths occurred within the first 2 weeks of the episode.[72] Moreover, the median survival of individuals with more than 1.25 episodes of peritonitis per patient year was reported to be 30 months shorter when compared to those with fewer episodes.[73]

However, the most compelling evidence was provided by a recent study by Lam and colleagues. They demonstrated that in many patients, serum C-reactive protein remains elevated up to 6 weeks despite successful treatment of episodes of peritonitis (mean serum C-reactive protein at baseline 8.8 mg/l and at 6 weeks 40.9 mg/l).[74] Individuals with serum C-reactive protein > 3.0 mg/l at 6 weeks after an episode of peritonitis had a greater risk for death. Thus, the persistent inflammation following acute episodes of intraperitoneal infection may increase CV mortality of PD patients. This provides another strong rationale for the institution of measures to reduce the peritonitis rate of PD patients.

## Hyperhomocysteinemia

Progressive loss of renal function is associated with increase in plasma homocysteine levels and the median homocysteine levels of dialysis patients are two to threefold higher than that among individuals in the general population. Epidemiologic studies in the general population have demonstrated a direct association between plasma homocysteine levels and all-cause and CV mortality, and extrapolating these data, it has been argued that hyperhomocysteinemia may be one of the reasons for the high CV risk seen of dialysis patients. However, epidemiologic studies show that low rather than high homocysteine levels predict risk for death. This paradoxical is not unlike that seen with cholesterol levels and is probably secondary to the confounding influence of protein-energy wasting.[75] Moreover, two large randomized controlled trials – one in dialysis and non-dialysis dependent CKD patients (HOST) and the other in those with a functioning renal transplant but low estimated glomerular filtration rate (FAVORIT) – have failed to demonstrate any survival advantage in individuals treated aggressively with a combination of vitamin B6, folate, and B12.[76] These data are consistent with the lack of benefit with homocysteine lowering in the general population. Unless more data are reported, high-dose vitamin B administration cannot be recommended at this time. However, replacement doses of vitamins are still needed for dialysis patients to compensate for the loss of small amounts of water-soluble vitamins with dialysis therapy.

## Conclusion

Dialysis patients, including those treated with PD, have a high risk for mortality, particularly from CV causes. Over the last decade, the mortality rates of patients treated with PD have improved in the United States, whereas the 1-year mortality of HD patients has remained unchanged.[77–78] However, the mortality rates still remain unacceptably high. Two randomized controlled trials have demonstrated that increasing small solute clearances within the range achievable in clinical practice has no effect on patient morbidity and mortality.[79,80] Furthermore, as discussed above, there is limited direct evidence on which one can base recommendations for therapies that reduce CV risk. Up until such evidence is available, one has to use clinical judgment to optimize the management of traditional and non-traditional risk factors considered to be important in the genesis of vascular disease in patients with ESRD.
